# Suicide rates around Chinese and western valentine’s days in Taiwan: The roles of gender and marriage status

**DOI:** 10.1371/journal.pone.0332652

**Published:** 2025-10-15

**Authors:** Cheng-Fang Yen, Vincent Chin-Hung Chen, Hsing-Ying Ho, Ying-Yeh Chen, Dian-Jeng Li, Ray C. Hsiao, Yi-Lung Chen

**Affiliations:** 1 Department of Psychiatry, Kaohsiung Medical University Hospital, Kaohsiung Medical University, Kaohsiung, Taiwan; 2 Department of Psychiatry, School of Medicine, College of Medicine, Kaohsiung Medical University, Kaohsiung, Taiwan; 3 College of Professional Studies, National Pingtung University of Science and Technology, Pingtung, Taiwan; 4 School of Medicine, Chang Gung University, Taoyuan, Taiwan; 5 Department of Psychiatry, Chiayi Chang Gung Memorial Hospital, Chiayi, Taiwan; 6 Department of Healthcare Administration, Asia University, Taichung, Taiwan; 7 Taipei City Psychiatric Centre, Taipei City Hospital, Taipei City, Taiwan; 8 Institute of Public Health, School of Medicine, National Yang Ming Chiao Tung University, Taipei City, Taiwan; 9 Graduate Institute of Medicine and School of Medicine, College of Medicine, Kaohsiung Medical University, Kaohsiung, Taiwan; 10 Department of Addiction Science, Kaohsiung Municipal Kai-Syuan Psychiatric Hospital, Kaohsiung, Taiwan; 11 Department of Nursing, Meiho University, Pingtung, Taiwan; 12 Department of Psychiatry and Behavioral Sciences, University of Washington School of Medicine, Seattle, Washington, United States of America; 13 Department of Psychiatry, Seattle Children’s, Seattle, Washington, United States of America; Caleb University, NIGERIA

## Abstract

**Background:**

The suicide risk on Chinese Valentine’s Day and its potential risk factors have not been examined. This study assessed whether the suicide rates around Chinese and Western Valentine’s Days differed from the rest of the year in Taiwan, and the roles of various genders and marital statuses.

**Methods:**

This study analyzed daily suicide data from Taiwan’s Cause of Death Statistics between January 2012 and December 2022. We compared the suicide rate of each day in the week before and after Chinese and Western Valentine’s Days with those of the remainder of the year using Quasi-Poisson regression models stratified by gender and marital status. We then performed a moderation analysis to explore whether the effect of Valentine’s Day on suicide differed by gender.

**Results:**

Married women reported a higher suicide risk on the third day after Chinese Valentine’s Day than married men did. Although a similar trend was observed in Western Valentine’s Day between married women and men, it did not reach statistical significance.

**Conclusion:**

Suicide rates for certain days in the week before or after Chinese and Western Valentine’s Days were different from other days of the year, and these differences were gender- and marital status-specific.

## Introduction

Suicide is a global health concern, and investigating high-risk timeframes for suicide can provide valuable insights into optimal periods for expanding the availability of health-care services. Christmas and New Year’s Day are the most frequently examined holidays in terms of suicide rates. Studies have demonstrated a dip in suicide rates before or during Christmas and a peak after New Year’s Day [1–9]. Several hypotheses have been proposed to explain this phenomenon. For example, Durkheim posited that increased social integration during Christmas reduces the acceptability of suicide and enhances social support, thereby diminishing the risk of suicide [10,11]. Gabennesch [12] introduced the “broken-promise theory,” which suggests that major holidays like Christmas create expectations of improvement that temporarily lower the risk of suicide; however, unmet expectations can lead to frustration and disappointment, potentially triggering suicidal behaviors after the holidays [1,2]. Moreover, major holidays are often accompanied by stressors such as increased alcohol consumption, disrupted sleep patterns, financial burdens, and family conflicts, all of which can heighten the risk of suicide [1,2].

Compared with the suicide risk associated with Christmas and New Year’s Day, the link between Valentine’s Day and suicidal behavior is less well-studied. Traditionally associated with love and romance, Valentine’s Day has been increasingly commercialized, as evidenced by the popularization of gift-giving to express affection [13]. Disappointment from unmet expectations, the inability to meet a partner’s expectations, unhappy relationships, or unrequited love can lead to distress around Valentine’s Day [14]. If Valentine’s Day increases the risk of suicide, intervention programs should be developed to support and provide coping strategies for vulnerable individuals. Several studies have examined the correlations between Valentine’s Day and suicide and parasuicide; however, the results are mixed. Davenport and Birtle [15] found an increase in non-fatal deliberate self-harm on Valentine’s Day, whereas other studies have not found a significant increase in suicide or deliberate self-harm on Valentine’s Day [16–18].

Several aspects of the relationship between Valentine’s Day and suicide warrant further investigation. First, in traditional Chinese culture, the 7th day of the 7th lunar month—Chinese Valentine’s Day—has a millennia-long history but is observed similarly to the Western Valentine’s Day in modern Chinese-culture societies, with long-distance lovers often exchanging gifts. Given this cultural parallel, the relationship between Chinese Valentine’s Day and suicide risk deserves exploration. Second, studies have compared the number of suicides or parasuicides on Valentine’s Day with those on other days [15–17]. According to the “broken-promise theory” [12], individuals may experience a dynamic process of anticipation and disappointment around Valentine’s Day. Therefore, studies should examine changes in the suicide rate in the days before and after Valentine’s Day, not just on Valentine’s Day. Third, research has shown gender differences in the aftermath of New Year’s Day, with a more pronounced recovery in men’s suicide rates [19], and a longer duration of distress associated with Valentine’s Day among women (Lange et al., 2022). It has been hypothesized that women care more about the Valentine’s Day experience than men do, whereas men are more likely than women to feel pressure to meet their partner’s expectations on Valentine’s Day [14]. Moreover, a study in South Korea demonstrated that married men are a vulnerable population for increased suicide rates on lunar New Year’s Day and Thanksgiving Day [8]. Individuals of various marital statuses may have different attitudes, expectations, and distress on Valentine’s Day. Whether the risk of suicide on Valentine’s Day varies among individuals of different genders and marital statuses needs to be examined.

The present population-based study assessed whether suicide rates around Chinese and Western Valentine’s Days differed from the rest of the year. We hypothesized that suicide rates around Chinese and Western Valentine’s Days differed from the rest of the year. We also hypothesized that the correlations between Valentine’s Days and suicide rates varied among individuals of different genders and marital statuses.

## Methods

### Suicide data

Daily suicide data were sourced from Taiwan’s national Cause-of-Death database, which was accessed on July 1, 2023. Suicides were identified using International Classification of Diseases, 10th Revision (ICD-10) codes X60–X84. We extracted daily suicide counts from 2012 to 2022. Along with the cause of death, demographic information such as gender and marital status (categorized as single, married, divorced, widowed, and unknown) was also available. We included only decedents aged 18 and older, excluding those with unknown marital status from the analysis. This study was approved by the Institutional Review Board of Taipei City Hospital Research Ethics Committee (TCHIRB-11012016). Informed consent is not required as this study used existing death records.

### Chinese and Western Valentine’s Day

The dates for Chinese Valentine’s Day from 2012 to 2022 are as follows: August 23, 2012; August 13, 2013; August 2, 2014; August 20, 2015; August 9, 2016; August 28, 2017; August 17, 2018; August 7, 2019; August 25, 2020; August 14, 2021; and August 4, 2022. In contrast, Western Valentine’s Day consistently falls on February 14 each year.

### Effect modifier

In this study, we examined whether gender modified the association between the occurrence of Chinese and Western Valentine’s Days and suicide, and whether this gender-based effect modification varied across different marital status groups. Marital status was conceptualized as a second-order effect modifier, influencing the extent to which gender modified the relationship between Valentine’s Days and suicide risk. Information on gender and marital status of individuals who died by suicide was obtained from Taiwan’s National Cause-of-Death Database.

### Statistical analysis

We first reported the average number of daily suicide cases across the entire year, as well as during the weeks before and after Chinese and Western Valentine’s Day, along with their corresponding standard errors. The standard errors of the average daily suicide counts were calculated as the sample standard deviation divided by the square root of the number of observed days. Furthermore, to evaluate whether suicide rates during the two-week periods surrounding Chinese and Western Valentine’s Days differed from those during the remainder of the year, we applied Quasi-Poisson regression models for each calendar year and for the entire study period from 2012 to 2022. The Quasi-Poisson regression model was applied to account for overdispersion—that is, the violation of the Poisson model’s assumption that the mean equals the variance, by introducing a dispersion parameter to adjust the variance estimation. We adjusted for potential confounding effects of time by including categorical variables for year and month. Incidence rate ratios (IRRs) and 95% confidence intervals (CIs) were calculated to characterize these associations. An IRR greater than 1 indicates an increased suicide risk, while a 95% CI not containing the null value signifies statistical significance. A two-tailed p-value was also reported.

We further compared the suicide rate of each day in the week before and after the Chinese and Western Valentine’s Days with that of the remainder of the year. These analyses were initially conducted in overall sample, then stratified by gender and marital status. We then performed a moderation analysis to explore whether the effect of Chinese Valentine’s Day on suicide differed by gender, including an interaction term of gender by the occurrence of Chinese Valentine’s Day to indicate differential effects. A statistically significant interaction would indicate that the association between Chinese Valentine’s Day and suicide risk differs by gender when compared to other times of the year. Furthermore, we examined whether this gender-based effect modification varied across different marital status groups. Finally, we conducted similar analyses for Western Valentine’s Day to investigate whether a comparable or distinct pattern emerged.

## Results

### Comparison of average daily suicide rates

Table 1 summarizes annual suicide counts and the average number of daily suicide deaths during the two-week periods surrounding Western and Chinese Valentine’s Days, as well as during the remainder of the year, for each year from 2012 to 2022. Overall, the annual and average number of suicide cases was highest during the period from 2017 to 2019.

**Table 1:** Annual Counts and average daily of Suicide Cases During the Week Before and After Chinese and Western Valentine’s Day and other times from 2012 to 2022.

Year	Annual Counts				Average number of daily counts			
	Total	Week before and after Western Valentine’s Day	Week before and after Chinese Valentine’s Day	Other time	Total	Week before and after Western Valentine’s Day	Week before and after Chinese Valentine’s Day	Other time
	N	n (%)	n (%)	n (%)	Mean (SE)	Mean (SE)	Mean (SE)	Mean (SE)
2012	3221	142 (4.4)	105 (3.3)	2974 (92.3)	8.80 (0.19)	9.47 (0.82)	7.00 (0.72)	8.85 (0.20)
2013	2800	116 (4.1)	119 (4.3)	2565 (91.6)	7.67 (0.16)	7.73 (0.71)	7.93 (0.67)	7.66 (0.17)
2014	3367	149 (4.4)	118 (3.5)	3100 (92.1)	9.22 (0.16)	9.93 (0.75)	7.87 (0.72)	9.25 (0.17)
2015	2863	104 (3.6)	125 (4.4)	2634 (92.0)	7.84 (0.23)	6.93 (0.78)	8.33 (0.78)	7.86 (0.24)
2016	3388	161 (4.8)	140 (4.1)	3087 (91.1)	9.26 (0.19)	10.73 (1.08)	9.33 (1.18)	9.19 (0.20)
2017	3836	163 (4.2)	156 (4.1)	3517 (91.7)	10.51 (0.19)	10.87 (0.93)	10.40 (0.85)	10.5 (0.20)
2018	3826	139 (3.6)	157 (4.1)	3530 (92.3)	10.48 (0.18)	9.27 (1.24)	10.47 (0.91)	10.54 (0.19)
2019	3809	167 (4.4)	153 (4.0)	3489 (91.6)	10.44 (0.17)	11.13 (1.02)	10.20 (0.54)	10.41 (0.18)
2020	3601	161 (4.5)	144 (4.0)	3296 (91.5)	9.84 (0.18)	10.73 (0.94)	9.60 (0.65)	9.81 (0.19)
2021	3546	142 (4.0)	156 (4.4)	3248 (91.6)	9.72 (0.17)	9.47 (0.85)	10.4 (0.84)	9.70 (0.18)
2022	3737	146 (3.9)	163 (4.4)	3428 (91.7)	10.24 (0.17)	9.73 (0.86)	10.87 (0.89)	10.23 (0.17)

Standard error = SE

Suicide risks during the two-week periods surrounding Chinese and Western Valentine’s Days were generally comparable to those observed during other times of the year, with the exception of 2014 (Table 2), when a lower suicide risk was observed around Chinese Valentine’s Day compared to Western Valentine’s Day (IRR = 0.78, 95% CI = 0.64–0.96, P-Value of 0.019).

**Table 2:** Comparison of daily suicide cases during the week before and after Chinese or Western Valentine’s Day, compared to other times, from 2012 to 2022.

Year	Western Valentine’s Day vs. Other time	Chinese Valentine’s Day vs. Other time	Chinese Valentine’s Day vs. Western Valentine’s Day
	IRR (95% CI)	IRR (95% CI)	IRR (95% CI)
2012^a^	0.91 (0.73-1.15)	0.83 (0.64-1.07)	0.91 (0.64-1.28)
2013^a^	1.16 (0.88-1.53)	1.08 (0.84-1.40)	0.94 (0.64-1.37)
2014^a^	0.92 (0.73-1.16)	0.78 (0.64-0.96)	0.85 (0.62-1.15)
2015^a^	0.92 (0.70-1.21)	0.91 (0.71-1.15)	0.99 (0.68-1.42)
2016^a^	1.27 (1.00-1.61)	1.01 (0.80-1.27)	0.79 (0.57-1.10)
2017^a^	1.11 (0.88-1.40)	0.89 (0.74-1.07)	0.80 (0.60-1.08)
2018^a^	0.91 (0.71-1.15)	1.00 (0.80-1.24)	1.10 (0.80-1.52)
2019	0.94 (0.76-1.17)	1.01 (0.82-1.25)	1.08 (0.79-1.46)
2020^a^	1.10 (0.87-1.38)	0.92 (0.74-1.13)	0.84 (0.61-1.14)
2021^a^	0.88 (0.70-1.11)	1.17 (0.93-1.47)	1.33 (0.96-1.85)
2022^a^	0.97 (0.77-1.23)	1.04 (0.87-1.25)	1.07 (0.79-1.44)
All^b^	1.00 (0.93-1.07)	0.95 (0.89-1.01)	0.95 (0.87-1.05)

IRR = incidence rate ratio, CI = confidence interval. Standard error = se.

^a^These analyses were conducted with adjustment for month.

^b^These analyses were conducted with adjustment for month and year.

### Gender and marital status differences in suicide risk around Chinese Valentine’s Day

**Fig 1:**
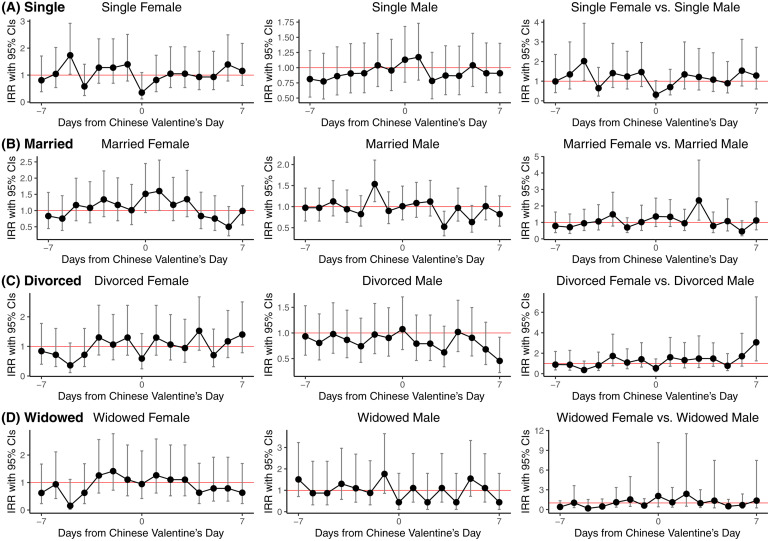
Suicide risk during the Chinese Valentine’s Day, compared to other times of the year in Taiwan from 2012 to 2022, stratified by gender and marital status.

We further compared the suicide rate on each day within the week before and after Chinese Valentine’s Day with the rates during the rest of the year, utilizing a Quasi-Poisson regression model. In the overall sample, a significantly higher suicide risk was observed on the second day preceding Chinese Valentine’s Day (IRR = 1.20, 95% CI = 1.01–1.44, P-Value of 0.041) compared to other days from 2012 to 2022 (S1 Table). Additionally, we conducted effect modification analyses stratified by gender (with man served as the reference group) and marital status (S2 Table and Fig 1). Among single individuals, a statistically significant higher suicide risk was found on the fifth day preceding Chinese Valentine’s Day for women (IRR = 1.74, 95% CI = 1.03–2.92, P-Value of 0.038) but not for men (IRR = 0.86, 95% CI = 0.55–1.34, P-Value of 0.509); moderation analysis further confirmed this gender difference (IRR = 2.02, 95% CI = 1.03–3.95, P-Value of 0.040). For married individuals, women exhibited an increased suicide risk on the first day following Chinese Valentine’s Day (IRR = 1.60, 95% CI = 1.00–2.56, P-Value of 0.049). In contrast, married men showed an elevated suicide risk on the second day before Chinese Valentine’s Day (IRR = 1.53, 95% CI = 1.12–2.11, P-Value of 0.008) but a reduced risk on the third day afterward (IRR = 0.52, 95% CI = 0.31–0.89, P-Value of 0.008). Moderation analysis indicated that married women had a significantly higher suicide risk than married men on the third day after Chinese Valentine’s Day (IRR = 2.33, 95% CI = 1.14–4.78, P-Value of 0.021). Among divorced men, a significantly lower suicide risk was observed on the seventh day after Chinese Valentine’s Day (IRR = 0.46, 95% CI = 0.23–0.92, P-Value of 0.028), also supported by moderation analysis. Finally, suicide risk around Chinese Valentine’s Day was comparable to other days for both widowed women and men based on the moderation analysis (IRR = 3.06, 95% CI = 1.25–7.51, P-Value of 0.014).

### Gender and marital status differences in suicide risk around Western Valentine’s Day

**Fig 2:**
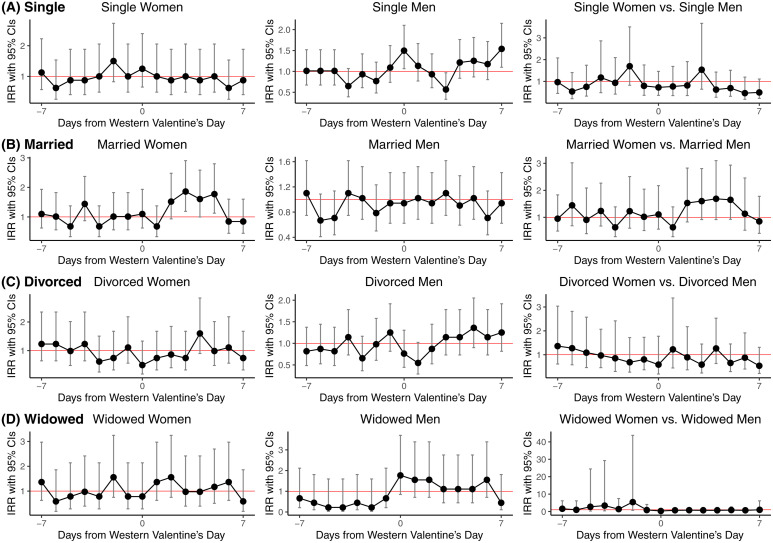
Suicide risk during the Western Valentine’s Day, compared to other times of the year in Taiwan from 2012 to 2022, stratified by gender and marital status.

We conducted similar analyses for Western Valentine’s Day compared with the rates during the rest of the year. Overall, a significantly higher suicide risk (IRR = 1.22, 95% CI = 1.02–1.47, P-Value of 0.027) was observed on the fifth day following Western Valentine’s Day compared to other days of the year from 2012 to 2022 (S3 Table). In further effect modification analyses using man as the reference group (S4 Table and Fig 2), married women showed an elevated suicide risk from the third to fifth days following Western Valentine’s Day (IRR = 1.86, 95% CI = 1.19–2.91, P-Value of 0.006). While moderation analysis revealed a similar trend, it did not achieve statistical significance (IRR = 1.60, 95% CI = 0.92–2.81, P-Value of 0.098). Finally, based on stratified analyses and moderation analyses, suicide risks around Western Valentine’s Day were comparable to other periods for single, divorced, and widowed individuals, regardless of gender.

## Discussion

The present study demonstrated that suicide rates on specific days around Chinese and Western Valentine’s Day differ from other days of the year, with these differences being gender and marital status-specific. Although suicide risk varied on these specific days, no corresponding differences were observed when analyses were conducted at the yearly level. A possible explanation is that these differences in risk persist only for a few days, so summing or averaging over the entire period surrounding Valentine’s Day dilutes the effect. This interpretation is supported by our findings, which indicate that not all days in this period exhibited statistically significant differences in suicide risk compared with other days of the year. Notably, married women exhibited a higher suicide rate on the third day after Chinese Valentine’s Day and between the third and fifth days after Western Valentine’s Day. These periods of increased risk may reflect unmet expectations related to Valentine’s Day, a time traditionally associated with romance and love. A possible reason is that women, more so than men, tend to value the expression of love through gestures such as dining out, gift-giving, or verbal affirmations on Valentine’s Day [20]. When these expectations are not met, married women may feel a strong sense of disappointment that can increase the risk of suicide. Women in poor marital relationships may also feel more pressure than usual on Valentine’s Day, consequently increasing the risk of suicide.

The present study found that married men had an increased suicide rate on the second day before Chinese Valentine’s Day but a lower suicide rate on the third day after Chinese Valentine’s Day. Because men are still expected to play the giver’s role in the Valentine’s Day ritual, married men may feel pressure about the upcoming Chinese Valentine’s Day, such as feeling that they should conform to societal expectations of gift-giving on a commercialized Chinese Valentine’s Day. These stresses are relieved after Chinese Valentine’s Day, and thus the suicide risk decreases in married men.

The present study found that single women, but not single men had a higher risk of suicide on the fifth day preceding Chinese Valentine’s Day, indicating that the upcoming Valentine’s Day makes single women feel stressed about not having a partner. The present study also found that divorced men had a lower suicide rate on the seventh day after Chinese Valentine’s Day compared with divorced women, indicating that divorced men and women may have different feelings about the end of Chinese Valentine’s Day.

These findings underscore the importance of developing interventions that address the unique stressors Valentine’s Day introduces and enhance support and coping mechanisms tailored to different demographic groups. Further research is necessary to delve deeper into the dynamics between Valentine’s Day and changes in suicide rates, particularly to understand the gender- and marital status-specific nuances observed around Chinese Valentine’s Day, which were not as pronounced during Western Valentine’s Day.

There are several limitations to this study. Firstly, despite obtaining suicide rates across various gender and marital status groups, we lacked specific information regarding the participants’ feelings about Valentine’s Day and their perceptions of their current marital status. Secondly, factors such as mental disorders and economic capacity that might influence or mediate the correlation between Valentine’s Day experiences and suicide were not determined in this study. Thirdly, this was an observational study, limiting the causal inferences that can be drawn from our results. Fourthly, some common potential factors, such as age, may also serve as effect modifiers which have influence on the association between the occurrence of Western or Chinese Valentine’s Day and suicide. Research may benefit from further stratified analyses to assess whether the impact of these culturally significant dates on suicide risk varies across different age groups. Finally, our findings indicated that significant suicide risks occurred on specific isolated days rather than over a continuous period surrounding Valentine’s Day. We did not assess whether these daily risks recurred annually during the study period (2012–2022) because a year-by-year analysis was statistically unreliable; stratifying by year, day, and demographic subgroup yielded daily suicide event counts that were too low—sometimes zero—to produce reliable estimates. Therefore, we analyzed the entire study period to obtain relatively reliable estimates. Future research employing larger, multi-national datasets is warranted to confirm these specific daily risk patterns.

## Conclusion

The present study found that the suicide rates for certain days in the week before or after Chinese and Western Valentine’s Day were different from other days of the year, and these differences were gender and marital status specific. The findings highlight the need for targeted interventions considering gender and marital status in suicide prevention strategies.

## Supporting information

S1 Table Suicide risk during the Chinese Valentine’s Day, compared to other times of the year in Taiwan from 2012 to 2022 in whole sample. (DOCX)

S2 Table Suicide risk during the Chinese Valentine’s Day, compared to other times of the year in Taiwan from 2012 to 2022, stratified by gender and marital status. (DOCX)

S3 Table Suicide risk during the Western Valentine’s Day, compared to other times of the year in Taiwan from 2012 to 2022. (DOCX)

S4 Table Suicide risk during the Western Valentine’s Day, compared to other times of the year in Taiwan from 2012 to 2022, stratified by gender and marital status. (DOCX)
