# Supplementary material for: Suicide rates around Chinese and western valentine’s days in Taiwan: The roles of gender and marriage status
Source: PLoS One. 2025 Oct 15;20(10):e0332652. doi: 10.1371/journal.pone.0332652 (PMC12527142; doi:10.1371/journal.pone.0332652)
Supplement: S1 Table — (DOCX) [file pone.0332652.s001.docx]

S1Table. Suicide risk during the Chinese Valentine's Day, compared to other times of the year in Taiwan from 2012 to 2022 in whole sample

| Time during Valentine's Day | Overall sample | |
| --- | --- | --- |
|  | IRR (95CI) | P-value |
| -7 | 0.895 (0.730-1.096) | 0.281 |
| -6 | 0.857 (0.697-1.055) | 0.146 |
| -5 | 0.969 (0.797-1.179) | 0.754 |
| -4 | 0.885 (0.722-1.086) | 0.243 |
| -3 | 0.997 (0.822-1.210) | 0.978 |
| -2 | 1.202 (1.007-1.435) | 0.041 |
| -1 | 1.044 (0.863-1.263) | 0.657 |
| 0 | 0.988 (0.813-1.201) | 0.904 |
| 1 | 1.119 (0.930-1.345) | 0.233 |
| 2 | 0.960 (0.788-1.170) | 0.687 |
| 3 | 0.839 (0.679-1.036) | 0.103 |
| 4 | 0.940 (0.770-1.147) | 0.541 |
| 5 | 0.856 (0.695-1.054) | 0.143 |
| 6 | 0.912 (0.745-1.116) | 0.371 |
| 7 | 0.845 (0.686-1.041) | 0.114 |

IRR = incidence rate ratio, CI = confidence interval. The analyses were conducted with adjustment for month and year.
