# Supplementary material for: Suicide rates around Chinese and western valentine’s days in Taiwan: The roles of gender and marriage status
Source: PLoS One. 2025 Oct 15;20(10):e0332652. doi: 10.1371/journal.pone.0332652 (PMC12527142; doi:10.1371/journal.pone.0332652)
Supplement: S2 Table — (DOCX) [file pone.0332652.s002.docx]

S2 Table. Suicide risk during the Chinese Valentine's Day, compared to other times of the year in Taiwan from 2012 to 2022, stratified by gender and marital status

| Time during Valentine's Day | Women | | Men | | Women vs. Men | |
| --- | --- | --- | --- | --- | --- | --- |
|  | IRR (95CI) | P-value | IRR (95CI) | P-value | IRR (95CI) | P-value |
| *Single* |  |  |  |  |  |  |
| -7 | 0.807 (0.381-1.708) | 0.575 | 0.813 (0.516-1.282) | 0.374 | 0.991 (0.416-2.360) | 0.984 |
| -6 | 1.041 (0.535-2.026) | 0.905 | 0.775 (0.485-1.238) | 0.286 | 1.345 (0.604-2.997) | 0.468 |
| -5 | 1.735 (1.030-2.924) | 0.038 | 0.861 (0.552-1.344) | 0.509 | 2.017 (1.032-3.946) | 0.040 |
| -4 | 0.578 (0.239-1.402) | 0.226 | 0.904 (0.585-1.396) | 0.649 | 0.640 (0.241-1.700) | 0.371 |
| -3 | 1.278 (0.696-2.347) | 0.430 | 0.909 (0.587-1.406) | 0.667 | 1.409 (0.679-2.926) | 0.358 |
| -2 | 1.278 (0.696-2.347) | 0.430 | 1.038 (0.690-1.564) | 0.857 | 1.233 (0.603-2.520) | 0.566 |
| -1 | 1.397 (0.777-2.512) | 0.263 | 0.956 (0.623-1.467) | 0.837 | 1.467 (0.725-2.969) | 0.286 |
| 0 | 0.349 (0.112-1.094) | 0.071 | 1.130 (0.760-1.679) | 0.546 | 0.310 (0.094-1.026) | 0.055 |
| 1 | 0.815 (0.382-1.737) | 0.596 | 1.173 (0.795-1.731) | 0.421 | 0.697 (0.303-1.603) | 0.396 |
| 2 | 1.048 (0.536-2.051) | 0.891 | 0.782 (0.488-1.253) | 0.307 | 1.345 (0.604-2.997) | 0.468 |
| 3 | 1.048 (0.536-2.050) | 0.891 | 0.869 (0.555-1.361) | 0.540 | 1.210 (0.551-2.661) | 0.635 |
| 4 | 0.928 (0.458-1.882) | 0.837 | 0.867 (0.555-1.354) | 0.530 | 1.076 (0.473-2.446) | 0.861 |
| 5 | 0.928 (0.458-1.882) | 0.837 | 1.040 (0.691-1.566) | 0.851 | 0.897 (0.402-1.998) | 0.790 |
| 6 | 1.393 (0.778-2.494) | 0.265 | 0.910 (0.588-1.408) | 0.672 | 1.537 (0.755-3.128) | 0.236 |
| 7 | 1.156 (0.614-2.176) | 0.653 | 0.907 (0.588-1.401) | 0.661 | 1.281 (0.602-2.723) | 0.520 |
| *Married* |  |  |  |  |  |  |
| -7 | 0.833 (0.445-1.559) | 0.567 | 0.974 (0.659-1.439) | 0.895 | 0.785 (0.378-1.629) | 0.516 |
| -6 | 0.752 (0.388-1.459) | 0.399 | 0.973 (0.658-1.440) | 0.893 | 0.707 (0.331-1.509) | 0.370 |
| -5 | 1.170 (0.685-1.998) | 0.565 | 1.123 (0.780-1.618) | 0.533 | 0.953 (0.505-1.798) | 0.881 |
| -4 | 1.087 (0.624-1.891) | 0.769 | 0.936 (0.628-1.395) | 0.745 | 1.061 (0.542-2.077) | 0.862 |
| -3 | 1.342 (0.811-2.222) | 0.252 | 0.823 (0.538-1.260) | 0.370 | 1.484 (0.779-2.829) | 0.230 |
| -2 | 1.175 (0.686-2.010) | 0.557 | 1.534 (1.118-2.105) | 0.008 | 0.697 (0.380-1.280) | 0.244 |
| -1 | 1.011 (0.565-1.807) | 0.971 | 0.898 (0.596-1.352) | 0.605 | 1.021 (0.510-2.043) | 0.954 |
| 0 | 1.516 (0.938-2.451) | 0.089 | 1.010 (0.686-1.487) | 0.961 | 1.361 (0.749-2.473) | 0.312 |
| 1 | 1.600 (1.002-2.557) | 0.049 | 1.085 (0.746-1.577) | 0.671 | 1.337 (0.749-2.388) | 0.326 |
| 2 | 1.179 (0.687-2.024) | 0.550 | 1.122 (0.776-1.622) | 0.540 | 0.953 (0.505-1.798) | 0.881 |
| 3 | 1.348 (0.811-2.238) | 0.249 | 0.524 (0.308-0.891) | 0.017 | 2.333 (1.137-4.784) | 0.021 |
| 4 | 0.834 (0.444-1.567) | 0.572 | 0.972 (0.656-1.439) | 0.887 | 0.785 (0.378-1.629) | 0.516 |
| 5 | 0.750 (0.386-1.458) | 0.397 | 0.635 (0.392-1.029) | 0.065 | 1.081 (0.481-2.426) | 0.851 |
| 6 | 0.500 (0.223-1.123) | 0.093 | 1.009 (0.687-1.484) | 0.962 | 0.454 (0.187-1.099) | 0.080 |
| 7 | 0.992 (0.558-1.764) | 0.977 | 0.822 (0.538-1.256) | 0.365 | 1.113 (0.550-2.252) | 0.765 |
| *Divorced* |  |  |  |  |  |  |
| -7 | 0.839 (0.396-1.777) | 0.647 | 0.932 (0.567-1.531) | 0.780 | 0.894 (0.367-2.175) | 0.804 |
| -6 | 0.715 (0.318-1.608) | 0.417 | 0.806 (0.474-1.372) | 0.427 | 0.875 (0.336-2.281) | 0.785 |
| -5 | 0.357 (0.114-1.116) | 0.077 | 0.979 (0.603-1.589) | 0.932 | 0.360 (0.106-1.231) | 0.103 |
| -4 | 0.715 (0.318-1.608) | 0.417 | 0.864 (0.517-1.445) | 0.577 | 0.817 (0.317-2.108) | 0.676 |
| -3 | 1.303 (0.709-2.393) | 0.394 | 0.741 (0.426-1.288) | 0.288 | 1.728 (0.773-3.863) | 0.182 |
| -2 | 1.066 (0.546-2.081) | 0.852 | 0.969 (0.596-1.575) | 0.900 | 1.081 (0.481-2.429) | 0.850 |
| -1 | 1.296 (0.703-2.388) | 0.405 | 0.903 (0.547-1.492) | 0.692 | 1.404 (0.651-3.030) | 0.387 |
| 0 | 0.589 (0.242-1.435) | 0.244 | 1.073 (0.676-1.704) | 0.766 | 0.538 (0.201-1.441) | 0.217 |
| 1 | 1.296 (0.703-2.388) | 0.405 | 0.790 (0.463-1.350) | 0.389 | 1.605 (0.728-3.540) | 0.241 |
| 2 | 1.060 (0.542-2.076) | 0.864 | 0.790 (0.463-1.350) | 0.389 | 1.313 (0.568-3.038) | 0.524 |
| 3 | 0.943 (0.463-1.918) | 0.871 | 0.621 (0.341-1.133) | 0.120 | 1.486 (0.597-3.698) | 0.395 |
| 4 | 1.526 (0.870-2.674) | 0.140 | 1.021 (0.637-1.637) | 0.931 | 1.475 (0.722-3.015) | 0.286 |
| 5 | 0.704 (0.312-1.586) | 0.397 | 0.908 (0.551-1.496) | 0.704 | 0.766 (0.299-1.960) | 0.578 |
| 6 | 1.173 (0.622-2.215) | 0.622 | 0.681 (0.383-1.209) | 0.189 | 1.702 (0.734-3.945) | 0.215 |
| 7 | 1.403 (0.786-2.505) | 0.253 | 0.456 (0.227-0.917) | 0.028 | 3.064 (1.251-7.505) | 0.014 |
| *Windowed* |  |  |  |  |  |  |
| -7 | 0.623 (0.232-1.674) | 0.348 | 1.513 (0.709-3.230) | 0.284 | 0.391 (0.114-1.337) | 0.134 |
| -6 | 0.938 (0.416-2.116) | 0.878 | 0.870 (0.322-2.356) | 0.785 | 1.025 (0.289-3.639) | 0.969 |
| -5 | 0.156 (0.022-1.116) | 0.064 | 0.870 (0.322-2.356) | 0.785 | 0.171 (0.019-1.530) | 0.114 |
| -4 | 0.625 (0.232-1.684) | 0.353 | 1.306 (0.575-2.962) | 0.523 | 0.456 (0.128-1.617) | 0.224 |
| -3 | 1.258 (0.617-2.566) | 0.528 | 1.096 (0.447-2.689) | 0.842 | 1.094 (0.357-3.350) | 0.875 |
| -2 | 1.415 (0.721-2.778) | 0.313 | 0.877 (0.323-2.381) | 0.796 | 1.538 (0.473-5.003) | 0.474 |
| -1 | 1.105 (0.515-2.369) | 0.797 | 1.765 (0.855-3.643) | 0.124 | 0.598 (0.216-1.653) | 0.322 |
| 0 | 0.947 (0.417-2.151) | 0.897 | 0.441 (0.109-1.793) | 0.253 | 2.051 (0.413-10.172) | 0.379 |
| 1 | 1.263 (0.617-2.585) | 0.523 | 1.103 (0.448-2.718) | 0.831 | 1.094 (0.357-3.349) | 0.875 |
| 2 | 1.105 (0.515-2.369) | 0.797 | 0.441 (0.109-1.793) | 0.253 | 2.392 (0.496-11.529) | 0.277 |
| 3 | 1.105 (0.515-2.369) | 0.797 | 1.103 (0.448-2.718) | 0.831 | 0.957 (0.303-3.021) | 0.940 |
| 4 | 0.630 (0.233-1.702) | 0.362 | 0.442 (0.109-1.791) | 0.253 | 1.367 (0.250-7.472) | 0.718 |
| 5 | 0.788 (0.323-1.921) | 0.6 | 1.547 (0.720-3.325) | 0.263 | 0.488 (0.155-1.541) | 0.222 |
| 6 | 0.788 (0.323-1.921) | 0.6 | 1.105 (0.451-2.710) | 0.827 | 0.684 (0.198-2.365) | 0.548 |
| 7 | 0.629 (0.233-1.695) | 0.359 | 0.443 (0.110-1.790) | 0.253 | 1.367 (0.250-7.472) | 0.718 |

IRR = incidence rate ratio, CI = confidence interval. The analyses were conducted with adjustment for month and year.
