# Supplementary material for: Suicide rates around Chinese and western valentine’s days in Taiwan: The roles of gender and marriage status
Source: PLoS One. 2025 Oct 15;20(10):e0332652. doi: 10.1371/journal.pone.0332652 (PMC12527142; doi:10.1371/journal.pone.0332652)
Supplement: S3 Table — (DOCX) [file pone.0332652.s003.docx]

S3 Table. Suicide risk during the Western Valentine's Day, compared to other times of the year in Taiwan from 2012 to 2022

| Time during Valentine's Day | Overall sample | |
| --- | --- | --- |
|  | IRR (95CI) | P-value |
| -7 | 1.036 (0.853-1.258) | 0.722 |
| -6 | 0.848 (0.685-1.049) | 0.128 |
| -5 | 0.810 (0.651-1.007) | 0.058 |
| -4 | 0.989 (0.811-1.206) | 0.911 |
| -3 | 0.829 (0.668-1.028) | 0.087 |
| -2 | 0.904 (0.735-1.112) | 0.339 |
| -1 | 1.036 (0.853-1.258) | 0.722 |
| 0 | 1.074 (0.887-1.299) | 0.466 |
| 1 | 0.942 (0.769-1.153) | 0.561 |
| 2 | 1.036 (0.853-1.258) | 0.722 |
| 3 | 1.026 (0.845-1.247) | 0.793 |
| 4 | 1.158 (0.963-1.393) | 0.118 |
| 5 | 1.224 (1.023-1.465) | 0.027 |
| 6 | 0.998 (0.819-1.216) | 0.986 |
| 7 | 1.064 (0.879-1.289) | 0.525 |

IRR = incidence rate ratio, CI = confidence interval. The analyses were conducted with adjustment for month and year.
