# Supplementary material for: Suicide rates around Chinese and western valentine’s days in Taiwan: The roles of gender and marriage status
Source: PLoS One. 2025 Oct 15;20(10):e0332652. doi: 10.1371/journal.pone.0332652 (PMC12527142; doi:10.1371/journal.pone.0332652)
Supplement: S4 Table — (DOCX) [file pone.0332652.s004.docx]

S4 Table. Suicide risk during the Western Valentine's Day, compared to other times of the year in Taiwan from 2012 to 2022, stratified by gender and marital status

| Time during Valentine's Day | Women | | Men | | Women vs. Men | |
| --- | --- | --- | --- | --- | --- | --- |
|  | IRR (95CI) | P-value | IRR (95CI) | P-value | IRR (95CI) | P-value |
| *Single* |  |  |  |  |  |  |
| -7 | 1.127 (0.571-2.226) | 0.731 | 1.012 (0.674-1.519) | 0.956 | 0.968 (0.451-2.077) | 0.934 |
| -6 | 0.626 (0.255-1.535) | 0.306 | 1.012 (0.674-1.519) | 0.956 | 0.538 (0.206-1.407) | 0.206 |
| -5 | 0.877 (0.408-1.884) | 0.736 | 1.012 (0.674-1.519) | 0.956 | 0.753 (0.325-1.743) | 0.508 |
| -4 | 0.877 (0.408-1.884) | 0.736 | 0.647 (0.392-1.069) | 0.089 | 1.177 (0.484-2.864) | 0.720 |
| -3 | 1.002 (0.488-2.055) | 0.996 | 0.931 (0.610-1.420) | 0.739 | 0.936 (0.418-2.094) | 0.871 |
| -2 | 1.503 (0.827-2.730) | 0.181 | 0.769 (0.484-1.221) | 0.265 | 1.699 (0.824-3.504) | 0.151 |
| -1 | 1.002 (0.488-2.055) | 0.996 | 1.093 (0.738-1.618) | 0.659 | 0.797 (0.362-1.756) | 0.574 |
| 0 | 1.252 (0.655-2.395) | 0.496 | 1.497 (1.066-2.103) | 0.020 | 0.727 (0.361-1.464) | 0.372 |
| 1 | 1.002 (0.488-2.055) | 0.996 | 1.133 (0.770-1.667) | 0.526 | 0.769 (0.350-1.688) | 0.512 |
| 2 | 0.877 (0.408-1.883) | 0.736 | 0.931 (0.610-1.420) | 0.739 | 0.819 (0.351-1.910) | 0.643 |
| 3 | 1.002 (0.488-2.055) | 0.996 | 0.566 (0.332-0.967) | 0.037 | 1.537 (0.644-3.668) | 0.333 |
| 4 | 0.877 (0.408-1.884) | 0.736 | 1.214 (0.835-1.764) | 0.310 | 0.628 (0.275-1.431) | 0.268 |
| 5 | 1.002 (0.488-2.055) | 0.996 | 1.254 (0.868-1.813) | 0.228 | 0.694 (0.319-1.512) | 0.358 |
| 6 | 0.626 (0.255-1.535) | 0.306 | 1.173 (0.803-1.715) | 0.409 | 0.464 (0.179-1.199) | 0.113 |
| 7 | 0.877 (0.408-1.883) | 0.736 | 1.538 (1.099-2.151) | 0.012 | 0.496 (0.221-1.111) | 0.088 |
| *Married* |  |  |  |  |  |  |
| -7 | 1.099 (0.624-1.934) | 0.745 | 1.099 (0.748-1.616) | 0.630 | 0.948 (0.490-1.831) | 0.873 |
| -6 | 1.014 (0.564-1.824) | 0.963 | 0.667 (0.410-1.086) | 0.104 | 1.441 (0.687-3.020) | 0.333 |
| -5 | 0.676 (0.332-1.376) | 0.280 | 0.707 (0.440-1.135) | 0.151 | 0.907 (0.394-2.088) | 0.819 |
| -4 | 1.437 (0.871-2.370) | 0.156 | 1.099 (0.748-1.616) | 0.630 | 1.239 (0.678-2.267) | 0.486 |
| -3 | 0.676 (0.332-1.376) | 0.280 | 1.021 (0.685-1.521) | 0.920 | 0.628 (0.284-1.388) | 0.250 |
| -2 | 1.014 (0.564-1.824) | 0.963 | 0.785 (0.500-1.233) | 0.293 | 1.225 (0.598-2.508) | 0.579 |
| -1 | 1.014 (0.564-1.824) | 0.963 | 0.942 (0.623-1.426) | 0.778 | 1.021 (0.510-2.043) | 0.954 |
| 0 | 1.099 (0.624-1.934) | 0.745 | 0.942 (0.623-1.426) | 0.778 | 1.106 (0.562-2.174) | 0.771 |
| 1 | 0.676 (0.332-1.376) | 0.280 | 1.021 (0.685-1.521) | 0.920 | 0.628 (0.284-1.388) | 0.250 |
| 2 | 1.521 (0.934-2.477) | 0.092 | 0.942 (0.623-1.426) | 0.778 | 1.531 (0.830-2.824) | 0.173 |
| 3 | 1.859 (1.190-2.905) | 0.006 | 1.099 (0.748-1.616) | 0.630 | 1.604 (0.916-2.807) | 0.098 |
| 4 | 1.606 (0.997-2.585) | 0.051 | 0.903 (0.592-1.378) | 0.636 | 1.686 (0.917-3.099) | 0.093 |
| 5 | 1.775 (1.125-2.798) | 0.014 | 1.021 (0.685-1.521) | 0.920 | 1.649 (0.927-2.933) | 0.089 |
| 6 | 0.845 (0.446-1.601) | 0.606 | 0.707 (0.440-1.135) | 0.151 | 1.134 (0.523-2.459) | 0.750 |
| 7 | 0.845 (0.446-1.601) | 0.606 | 0.942 (0.623-1.426) | 0.778 | 0.850 (0.406-1.780) | 0.667 |
| *Divorced* |  |  |  |  |  |  |
| -7 | 1.228 (0.643-2.348) | 0.534 | 0.815 (0.484-1.373) | 0.443 | 1.362 (0.611-3.035) | 0.450 |
| -6 | 1.228 (0.643-2.348) | 0.534 | 0.870 (0.525-1.442) | 0.589 | 1.277 (0.579-2.817) | 0.545 |
| -5 | 0.983 (0.479-2.015) | 0.962 | 0.815 (0.484-1.374) | 0.443 | 1.089 (0.461-2.573) | 0.845 |
| -4 | 1.228 (0.643-2.348) | 0.534 | 1.142 (0.731-1.783) | 0.560 | 0.973 (0.457-2.069) | 0.943 |
| -3 | 0.614 (0.251-1.505) | 0.287 | 0.652 (0.365-1.165) | 0.149 | 0.851 (0.300-2.418) | 0.762 |
| -2 | 0.737 (0.324-1.677) | 0.467 | 0.979 (0.606-1.579) | 0.929 | 0.681 (0.270-1.717) | 0.415 |
| -1 | 1.105 (0.560-2.182) | 0.773 | 1.250 (0.815-1.917) | 0.306 | 0.799 (0.369-1.730) | 0.570 |
| 0 | 0.491 (0.181-1.333) | 0.163 | 0.761 (0.444-1.304) | 0.320 | 0.584 (0.192-1.775) | 0.343 |
| 1 | 0.737 (0.324-1.677) | 0.467 | 0.544 (0.289-1.023) | 0.059 | 1.226 (0.445-3.376) | 0.694 |
| 2 | 0.860 (0.400-1.847) | 0.698 | 0.870 (0.525-1.442) | 0.589 | 0.894 (0.367-2.175) | 0.804 |
| 3 | 0.737 (0.324-1.677) | 0.467 | 1.142 (0.731-1.783) | 0.560 | 0.584 (0.235-1.448) | 0.245 |
| 4 | 1.597 (0.898-2.839) | 0.111 | 1.142 (0.731-1.783) | 0.560 | 1.265 (0.632-2.529) | 0.507 |
| 5 | 0.983 (0.479-2.015) | 0.962 | 1.359 (0.901-2.051) | 0.144 | 0.654 (0.294-1.451) | 0.296 |
| 6 | 1.105 (0.560-2.182) | 0.773 | 1.142 (0.731-1.783) | 0.560 | 0.875 (0.400-1.914) | 0.739 |
| 7 | 0.737 (0.324-1.677) | 0.467 | 1.250 (0.815-1.917) | 0.306 | 0.533 (0.217-1.310) | 0.170 |
| *Windowed* |  |  |  |  |  |  |
| -7 | 1.364 (0.627-2.970) | 0.434 | 0.664 (0.208-2.116) | 0.488 | 1.595 (0.412-6.177) | 0.499 |
| -6 | 0.585 (0.184-1.857) | 0.363 | 0.442 (0.108-1.810) | 0.256 | 1.025 (0.171-6.140) | 0.978 |
| -5 | 0.780 (0.284-2.137) | 0.629 | 0.221 (0.031-1.596) | 0.135 | 2.734 (0.305-24.471) | 0.368 |
| -4 | 0.975 (0.393-2.417) | 0.956 | 0.221 (0.031-1.596) | 0.135 | 3.418 (0.399-29.265) | 0.262 |
| -3 | 0.780 (0.284-2.137) | 0.629 | 0.442 (0.108-1.810) | 0.256 | 1.367 (0.250-7.472) | 0.718 |
| -2 | 1.559 (0.750-3.244) | 0.235 | 0.221 (0.031-1.596) | 0.135 | 5.468 (0.684-43.737) | 0.109 |
| -1 | 0.780 (0.284-2.137) | 0.629 | 0.664 (0.208-2.116) | 0.488 | 0.911 (0.204-4.078) | 0.903 |
| 0 | 0.780 (0.284-2.137) | 0.629 | 1.769 (0.846-3.700) | 0.130 | 0.342 (0.103-1.137) | 0.080 |
| 1 | 1.364 (0.627-2.971) | 0.434 | 1.548 (0.708-3.387) | 0.274 | 0.684 (0.239-1.953) | 0.477 |
| 2 | 1.559 (0.750-3.244) | 0.235 | 1.548 (0.708-3.387) | 0.274 | 0.781 (0.283-2.159) | 0.634 |
| 3 | 0.975 (0.393-2.416) | 0.956 | 1.106 (0.444-2.754) | 0.829 | 0.684 (0.198-2.365) | 0.548 |
| 4 | 0.975 (0.393-2.417) | 0.956 | 1.106 (0.444-2.754) | 0.829 | 0.684 (0.198-2.365) | 0.548 |
| 5 | 1.170 (0.508-2.695) | 0.713 | 1.106 (0.444-2.754) | 0.829 | 0.820 (0.250-2.692) | 0.744 |
| 6 | 1.364 (0.627-2.971) | 0.434 | 1.548 (0.708-3.387) | 0.274 | 0.684 (0.239-1.953) | 0.477 |
| 7 | 0.585 (0.184-1.858) | 0.363 | 0.442 (0.108-1.810) | 0.256 | 1.025 (0.171-6.143) | 0.978 |

IRR = incidence rate ratio, CI = confidence interval. The analyses were conducted with adjustment for month and year.
